# Discontinuity of psychiatric care for patients with schizophrenia, relation to previous psychiatric care and practice variation between providers: a retrospective longitudinal cohort study

**DOI:** 10.1186/s12888-021-03319-7

**Published:** 2021-06-29

**Authors:** Arnold P. M. van der Lee, Adriaan Hoogendoorn, Lieuwe de Haan, Aartjan T. F. Beekman

**Affiliations:** 1 Department Psychiatry Amsterdam University Medical Centre – location VUmc, Oldenaller 1, 1081 HJ Amsterdam, The Netherlands; 2 Department Psychiatry Amsterdam University Medical Centre – location AMC, Amsterdam, The Netherlands

**Keywords:** Schizophrenia, Continuity of care, Elective care, Practice variation, Logistic regression

## Abstract

**Background:**

Patients with schizophrenia need continuous integrated healthcare, but many discontinue their treatment, often experiencing adverse outcomes. The first objective of this study is to assess whether patient characteristics or treatment history are associated with discontinuity of psychiatric elective care. The second objective is to assess whether practice variation between providers of psychiatric care contributes to discontinuity of elective care.

**Methods:**

A large registry-based retrospective cohort of 9194 schizophrenia patients, who were included if they received elective psychiatric care in December 2014–January 2015. Logistic regression models were used to identify predictive factors of discontinuity of care. The dependent variable was the binary variable discontinuity of care in 2016. Potential independent predictive variables were: age, sex, urbanization, and treatment history in 2013–2014. Practice variation between providers was assessed, adjusting for the case mix of patients regarding their demographic and care utilization characteristics.

**Results:**

12.9% of the patients showed discontinuity of elective psychiatric care in the follow-up year 2016. The risk of discontinuity of care in 2016 was higher in younger patients (between age 18 and 26), patients with a history of receiving less elective psychiatric care, more acute psychiatric care, more quarters with elective psychiatric care without antipsychotic medication, or receiving no elective treatment at all. No evidence for practice variation between providers was found.

**Conclusions:**

Our findings show that the pattern of previous care consumption is an important prognostic factor of future discontinuity of elective care. We propose that previous care consumption can be used to design strategies to improve treatment retention and focus resources on those most at risk of dropping out.

## Background

Schizophrenia is a severe mental illness with often considerable impact on psychosocial functioning and quality of life [1–4]. Although most subjects diagnosed with schizophrenia show persistent vulnerability for a psychotic relapse, most patients are able to cope with possible persistent problems and about 30% of the patients experience a full clinical and functional recovery. The life expectancy of patients with schizophrenia is 15–25 years shorter, which is attributed to more suicides, severe side effects of antipsychotic medication and somatic comorbidities [5–15]. Patients require continuous, integrated healthcare which consists of community-oriented psychiatric and somatic care [1, 16–22]. Such treatment may help to prevent psychotic relapse and early mortality. Continuous integrated healthcare may also reduce costly acute interventions such as crisis treatment or hospitalization. Although all inhabitants in the Netherlands have access to quality healthcare for somatic and mental health problems and experience few financial barriers, not all patients with schizophrenia receive continuity of care [23, 24]. In two recent studies on large cohorts of patients with schizophrenia in the Netherlands, we found that over 3 years 73% of patients received continuity of care [21] and over 6 years 59% of patients [25] received continuity of care. Variation in continuity of care among patients can be attributed to patient characteristics, differences in the type of mental healthcare provided to the patient, and differences in the organization of the healthcare system. Knowledge about the factors driving discontinuity of healthcare may help implementing strategies to improve continuity of healthcare. For instance, by stratifying strategies which focus resources on those most at risk of dropping out. Or by improving continuity of treatment delivery in providers delivering suboptimal care.

We therefore examined the association between discontinuity of healthcare and (i) patient characteristics, (ii) patients’ treatment history, and (iii) mental healthcare providers. The object is to disentangle the factors hindering patients with schizophrenia from accessing continuity of integrated healthcare. Given a well-endowed health system with relatively few financial barriers to healthcare, our hypothesis is that discontinuity of care is determined by patient characteristics as well as treatment and provider characteristics.

## Methods

### Study design and patient selection

We examined discontinuity of care using a retrospective longitudinal cohort study design. Patients in the health insurance registry of the health insurer Zilveren Kruis were included in the current study if they had a diagnosis of schizophrenia in the period of 2013–2014 and if they received at least one day of elective psychiatric treatment in December 2014 or January 2015 as registered in the health insurance registry of Zilveren Kruis. Patients had to be insured by Zilveren Kruis for the whole study period 2013–2016 and had to be 18–69 years of age on 1-1-2015. Patients may have suffered their first episode of schizophrenia in 2013–2014, or may have had more episodes before 2013. Therefore, our sample is representative of the treated population with schizophrenia with an age of 18 to 69 years.

The year 2016 was used as the follow-up period to determine discontinuity of care. A claim for elective psychiatric treatment could last 365 days. Therefore, that episode of elective psychiatric treatment was finished before or in January 2016, followed by new treatment episodes according to the professional guidelines [26].

From this selection, only patients treated by providers with at least 50 patients in this dataset were included. All patients insured by Zilveren Kruis who met the selection criteria were included.

In summary, the inclusion criteria were:

- A DSM-IV diagnosis of schizophrenia in 2013–2014.
- Insured by Zilveren Kruis during the entire study period 2013–2016.
- 18–69 years of age on 1-1-2015.
- Receiving elective psychiatric treatment in December 2014 or January 2015, and
- treated by a provider with at least 50 patients with schizophrenia in the dataset.

### Data source: Dutch computerized health insurance registry data

All Dutch health insurance companies in the Netherlands keep thorough track of all claims and insurance data concerning the Dutch Health Insurance Act. The health insurance processes, including the data collection processes, are well regulated and monitored by the Dutch Healthcare Authority [27]. Zilveren Kruis is a major health insurance company which insures 30% of Dutch inhabitants.

In the Netherlands, healthcare is financed using the diagnoses treatment system (DBC). Every psychiatric treatment DBC-claim can last up to 365 days, except DBC-claims for crisis psychiatric treatment, which can last up to 28 days. The diagnoses of patients with schizophrenia are made by the psychiatrist who is treating the patient. The amount of psychiatric care patients received can be measured using the costs of DBC-claims: outpatient care is paid per minute and inpatient care by day and intensity of care provided.

The data collection and analyses of this study were performed under the strict privacy rules and regulations of the Dutch laws and the health insurance companies. Patients in the analysis could not be identified, therefore no informed consent or approval of a Medical Ethical Committee was necessary.

### Measures

#### Dependent variable: discontinuity of psychiatric care 2016

Discontinuity of psychiatric care was established when the patient neither received elective outpatient psychiatric care nor antipsychotic medication in at least one of all four quarters in 2016.

#### Independent variables

Discontinuity of psychiatric care might be affected by the severity of the symptoms of schizophrenia and side effects of antipsychotic medication. Patients with more severe symptoms or severe side effects often receive less elective and more acute psychiatric care. Therefore, a proxy for the severity of symptoms and side effects can be provided by information about the psychiatric care a patient has received in the past.

The prevalence of patients with schizophrenia is higher in urban areas compared to rural areas [28, 29]. Patients in urban areas may differ in severity of symptoms and side effects.

The healthcare system in the Netherlands is the same for all patients: they are covered by the same compulsory healthcare and health insurance system [30, 31]. Therefore, all inhabitants in the Netherlands have access to quality healthcare for somatic and mental health problems and experience few financial barriers to access their healthcare. Local variation in the level of continuity of care across mental healthcare institutes may be caused by case mix differences or by local practice variation.

Available independent variables were: age, sex, urbanization, and treatment history 2013–2014.

##### Age, sex, and urbanization

The patient characteristics that were available for our analysis were age, sex, and degree of urbanization on 1-1-2015. The 4-digit postal code of residence on 1-1-2015 was used to determine which of the five levels of urbanization [32] the patient lived in.

##### Treatment history 2013–2014

Treatment history 2013–2014 was measured in three ways:

*First:* For each of the eight quarters of the period 2013–2014, we labeled if a patient received elective psychiatric care, distinguishing four types of elective psychiatric treatment: (i) receiving both elective psychiatric treatment and antipsychotic medication, (ii) receiving elective psychiatric treatment only, (iii) receiving antipsychotic medication only, and (iv) receiving neither elective psychiatric treatment nor antipsychotic medication. For each of the four types of elective psychiatric treatment, the number of quarters was counted over the eight quarters in the period 2013–2014.

*Second:* The amount of psychiatric treatment a patient received over 2013–2014 was measured in two ways: (1) amount of elective psychiatric care, measured as total cost of elective psychiatric care; (2) amount of acute psychiatric care, measured as total cost of acute psychiatric care (crisis treatment or hospitalization). The costs of psychiatric care were calculated using average national prices in euros.

*Third:* If a patient received treatment for alcohol and/or opioid dependence was also measured. The reason is that alcohol and opioid dependence may complicate treatment and cooperation with treatment [33–37]. Alcohol and opioid dependence was measured in two ways: using diagnoses of addiction (DSM-IV) or the usage of medication which had ATC codes for treatment of alcohol and opioid dependence (N07BB, N07BC) [38, 39]. Alcohol and opioid dependence was divided into two categories: any alcohol or opioid dependence or none.

#### Allocation patients to psychiatric care providers

*First*, we allocated patients in the dataset to the healthcare provider delivering elective psychiatric treatment on 1-1-2015.

*Second*, if a patient did not receive elective psychiatric treatment on that day, then patients were allocated in the dataset to the last healthcare provider delivering elective psychiatric treatment in December 2014 or January 2015. Patients without elective psychiatric treatment in that period were not selected.

*Third*, only patients allocated to providers with at least 50 patients with schizophrenia in the dataset were included. We assumed that those providers had enough experience to provide appropriate continuous psychiatric care, which is often complicated for these patients.

### Overview study design

**Fig. 1:**
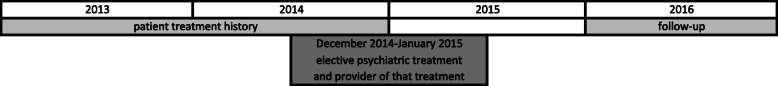
Study design

Figure 1 summarizes the different study periods. The entire study period was used as a first inclusion criterion: patients had to be insured at Zilveren Kruis during the entire period.

Over 2013–2014 patient treatment history was measured in three ways: the number of quarters patients received any of the four different types of elective psychiatric treatment, the amount of elective care received, the amount of acute care received, and treatment of alcohol or opioid dependence.

In the center is the two-month period around 1-1-2015 that was used as the second inclusion criterion: patients were included if they received some elective psychiatric treatment in that period. Those patients were allocated to the provider of that elective treatment.

The follow-up period (2016) was used to determine the outcome variable: discontinuity of care.

### Analysis

Stepwise logistic regression was used to identify predictive factors of discontinuity of care. The dependent variable was the binary variable discontinuity of care in 2016. Potential independent predictive variables were: age, sex, urbanization, treatment history 2013–2014 as measured: the number of quarters in which each of the four types of elective treatment was received, the amount of elective psychiatric care, the amount of acute psychiatric care, and alcohol or opioid dependence.

First, the univariate relation between each of the prognostic variables and discontinuity of care was analyzed using the log odds of the dependent variable discontinuity of elective psychiatric care. This was done to check the assumption of a linear relation of the continuous prognostic factors to discontinuity of care. In addition, collinearity between the prognostic factors was inspected using Pearson correlation coefficients.

Second, stepwise logistic regression was performed. A significance level of 0.05 was required for adding prognostic variables to the stepwise logistic regression model. The Akaike information criterion (AIC) was used to select the model [40]. Adjusted R^2^ (Nagelkerke R^2^), the Hosmer and Lemeshow goodness-of-fit test were calculated for the final selected model.

Third, variation between providers was assessed. First, the observed number of patients per provider who had discontinuity of care was measured. Then, for each provider, the expected number of patients with discontinuity of care was estimated from the final model of the stepwise logistic regression, thus adjusting for all relevant differences in available demographic and care characteristics. Next, for each provider, the standardized event ratio (SER) was calculated as the ratio of the observed number of cases to the expected number of cases [41]. A 95%-confidence interval was computed using the method of Rothman Greenland [42]. The null hypothesis that there was no significant practice variation was tested using a Chi-square test with k-1 degrees of freedom (k is the number of providers) [43].

All analyses were performed using SAS Enterprise guide 6.1 [SAS Institute Cary, NC, USA].

## Results

### Demographic and care characteristics of the study sample

Application of the inclusion criteria to the registry data of Zilveren Kruis resulted in a study sample of 9194 patients. The average age of the patients was 45.6. Women had an average age of 48, while men were 4 years younger with an average age of 44. Of all patients, 35.8% were women (Table 1). Over half of the patients (53.6%) lived in highly urbanized areas (compared to 23% of all Dutch inhabitants [32]). Diagnosis of alcohol or opioid dependence, or treatment for these disorders, was received by 5.5% of the patients.

**Table 1:** Demographic and treatment characteristics of the study sample (*n* = 9194)

*Demographic characteristics*				*Treatment characteristics*		
	*Category*	*number*	*%*		*Category*	*number*
Sex	Female	3294	35.8%	Elective care^a^	<=€3000	1227
					> €3000	7967
Age	18–22 years	211	2.3%			
	23–26 years	395	4.3%	Acute care^b^	€0	6691
	27–38 years	2155	23.4%		€0 - €10,000	1092
	39–61 years	5459	59.4%		€10,000 - €60,000	1088
	62–69 years	974	10.6%		€60,000 - €90,000	176
					> €90,000	147
Urbanization	High	4926	53.6%			
	Medium-high	2395	26.0%	Alcohol and opioid dependence		
	Medium	861	9.4%		yes	506
	Medium-low	638	6.9%			
	Low	373	4.1%	*Continuous variables*		*Mean*
				Nr of quarters^c^	Both E&A	5.97
					E only	1.01
					A only	0.50
					Neither E nor A	0.52

^a^ Amount of elective psychiatric care in the two-year period; ^b^ Amount of acute psychiatric care in the two-year period; ^c^ Number of quarters within the two-year period given the types of care: *E* Elective psychiatric care, *A* Antipsychotic medication

### Linearity assumption of logistic regression

Inspection of the univariate relation between discontinuity of care and the continuous demographic and care characteristics showed that age and amounts of elective and acute psychiatric care did not satisfy the linearity assumption. Therefore, categorical scales for these continuous prognostic variables were constructed, creating categories that matched the nonlinearity in the relationships with discontinuity. Thus, age was divided into five categories: 18–22 years, 23–26 years, 27–38 years, 39–61 years, and 62–69 years. Of these categories, category 39–61 contained the most patients (59.4%) and category 18–22 the least (2.3%, see Table 1). Besides the nonlinear relation to discontinuity, the amounts of both elective and acute psychiatric care showed spikes because of the structure of tariffs and very long thin tails. The amount of elective care was divided into two categories: up to €3000 (13.3%) and higher. The amount of acute care was divided over 5 categories: €0; €0 - € 10,000; €10,000 - €60,000; €60,000 - €90,000, and more than €90,000. Most patients (72.8%) did not receive acute psychiatric care.

Patients can have any of all possible combinations of the four types of elective care. On average patients’ usage of both elective psychiatric care and antipsychotic medication was the highest (5.97 out of 8 quarters in 2013–2014), while patients’ average usage of neither type of elective care was 0.52 out of 8 quarters. A large group of patients received optimal psychiatric care every quarter, but there was still much variation in which combinations of types of care patients received. We found that there was a large group of 4569 patients (50%) who received both elective psychiatric care and antipsychotic medication during all eight quarters. A small group of 316 patients (3%) received only elective psychiatric care during all eight quarters. Three patients received only antipsychotic medication during all eight quarters, and just one patient had no treatment at all over all quarters. Other patients received other combinations of the four types of care over the eight quarters.

### The relationship between demographic and care characteristics and discontinuity of care

Of all patients in the cohort, 12.9% had discontinuity of elective psychiatric care in 2016 (Table 2). This is less than an expected 16% based on the data from a previous cohort [21]. Difference in the prevalence of discontinuity between men (12.4%) and women (13.8%) was not statistically significant (*p* = 0.068).

**Table 2:** The relationship between demographic and care characteristics and discontinuity of care (*n* = 9194)

			Category level	Variable level
	Category	Discontinuity (%)	Odds Ratio (95%-CI)	Test results
*Total sample*		12.9%		
*Demographic characteristics*				
Sex	Male	12.4%	reference	χ^2^(1) = 3.32, p = 0.068
	Female	13.8%	1.12 (0.99, 1.27)	
Age	18–22	28.4%	3.18 (2.33, 4.34)	χ^2^(4) = 86.3, p < 0.001
	23–26	20.8%	2.10 (1.62, 2.71)	
	27–38	13.8%	1.29 (1.11, 1.49)	
	39–61	11.1%	reference	
	62–69	14.4%	1.34 (1.10, 1.64)	
Urbanization	high	13.5%	1.01 (0.82, 1.25)	χ^2^(4) = 6.31, *p* = 0.177
	medium-high	12.0%	0.89 (0.70, 1.12)	
	medium	13.4%	reference	
	medium-low	10.7%	0.77 (0.56, 1.06)	
	low	13.4%	1.00 (0.70, 1.43)	
*Psychiatric care characteristics*				
Elective care^a^	<=€3000	19.6%	1.82 (1.55, 2.12)	χ^2^(1) = 57.3, p < 0.001
	> €3000	11.9%	reference	
Acute care^b^	€0	11.0%	reference	χ^2^(4) = 86.6, p < 0.001
	€0 - €10,000	19.2%	1.92 (1.62, 2.28)	
	€10,000 - €60,000	15.7%	1.51 (1.26, 1.80)	
	€60,000 - €90,000	15.3%	1.46 (0.96, 2.22)	
	> €90,000	27.9%	3.13 (2.16, 4.52)	
Alcohol opioid dependence	No	13.0%	Reference	χ^2^(1) = 0.73, *p* = 0.392
	Yes	11.7%	0.89(0.67, 1.17)	
Number of quarters^c^	Both E and A		0.75 (0.73, 0.76)	*z* = −27.26, p < 0.001
	E only		1.36 (1.33, 1.39)	*z* = 24.89, *p* < 0.001
	A only		0.95 (0.90, 1.00)	*z* = −1.80, *p* = 0.073
	Neither E nor A		1.35 (1.31, 1.40)	*z* = 17.35, p < 0.001

^a^ Amount of elective care in the two-year period; ^b^ Amount of acute care in the two-year period; ^c^ Number of quarters within the two-year period receiving types of care: *E* Elective psychiatric care, *A* Antipsychotic medication;

Urbanization and alcohol and opioid dependency were not statistically significantly related to discontinuity (Table 2). Age, elective care, acute care, and number of quarters showed statistically significant relations with discontinuity. Discontinuity was highest in the age group of 18–22 (28.4%) and lowest in the age group of 39–61 (11.1%). Discontinuity was most prevalent (19.6%) in those receiving relatively little elective psychiatric care (costs of €3000 or less). Regarding acute psychiatric care, the highest prevalence of discontinuity (27.9%) was found in the small group receiving the most acute psychiatric care (costs of €90.000 or more). The lowest prevalence of discontinuity (11%) was found in the group without previous acute care.

As was expected, discontinuity was less likely in patients with a history of receiving both elective psychiatric care and antipsychotic medication during more quarters (odds ratio = 0.75, *p* < 0.001) (Table 2). The risk of discontinuity was higher in patients with a history of receiving either only elective psychiatric care without antipsychotic medication (odds ratio = 1.36, *p* < 0.001), or more quarters without any elective psychiatric care or antipsychotic medication (odds ratio = 1.35, p < 0.001).

The large group of 4569 patients who had optimal care (both elective psychiatric care and antipsychotic medication) in all eight quarters before follow-up showed very low discontinuity (5%) in 2016. The small group of 316 patients receiving elective psychiatric care but no medication during the preceding eight quarters were at a much higher risk of discontinuity (36.4%).

### Logistic regression

In our multivariate analyses, the adjusted R^2^ for the selected model was 17.8%. The prognostic factors for discontinuity in the model were: age, amount of elective psychiatric care, amount of acute psychiatric care, the number of quarters receiving both elective psychiatric care and antipsychotic medication, the number of quarters receiving antipsychotic medication only, and number of quarters receiving no elective psychiatric care nor antipsychotic medication (Table 3). Using other prognostic factors did not improve AIC.

**Table 3:** Regression parameters of the final logistic regression model obtained from stepwise forward variable selection (*n* = 9194)

	category	ln (OR)	SE	Chi2	*p*-value	OR (95%-CI)
*Demographic characteristics*						
Age	19–22	reference				
	23–26	0.108	0.114	0.899	0.343	1.11 (0.89, 1.39)
	27–38	− 0.105	0.071	2.175	0.140	0.90 (0.78, 1.04)
	39–61	−0.252	0.062	16.518	< 0.0001	0.78 (0.69, 0.88)
	62–69	−0.051	0.092	0.302	0.583	0.95 (0.79, 1.14)
*Psychiatric care characteristics*						
Amount of elective care^a^	<=€3000	reference				
	> €3000	−0.206	0.045	20.685	< 0.0001	0.81 (0.74, 0.89)
Amount of acute care^b^	€0	reference				
	€0 - €10,000	0.142	0.092	2.384	0.123	1.15 (0.96, 1.38)
	€10,000 - €60,000	0.026	0.095	0.074	0.786	1.03 (0.85, 1.24)
	€60,000 - €90,000	−0.233	0.182	1.640	0.200	0.79 (0.55, 1.13)
	> €90,000	0.300	0.164	3.336	0.068	1.35 (0.98, 1.86)
Number of quarters^c^	Both E and A	−0.317	0.013	598.308	< 0.0001	0.73 (0.71, 0.75)
	E only^d^	reference				
	A only	−0.323	0.034	91.707	< 0.0001	0.72 (0.68, 0.77)
	Neither E nor A	−0.055	0.022	6.039	0.014	0.95 (0.91, 0.99)
*Intercept*		0.376	0.118	10.16	0.001	1.46 (1.16, 1.84)

^a^ Costs of elective care in the two-year period; ^b^ Costs of acute care in the two-year period;^c^ Number of quarters within the two-year period receiving types of care: E = Elective psychiatric care; A = Antipsychotic medication; ^d^ One of the four variables was not entered into the model due to perfect multicollinearity, since for each patient these four variables add up to 8

### Practice variation

**Fig. 2:**
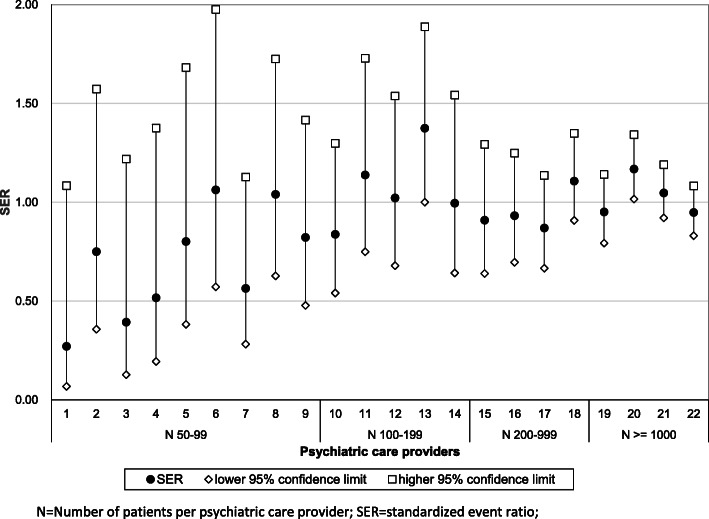
SER and 95% confidence intervals of 22 psychiatric care providers

The observed proportion of discontinuity and the expected proportion of discontinuity from the logistic regression model were combined to obtain an expected level of discontinuity for each provider. The standardized event ratios (SER) and the corresponding 95% confidence interval were calculated. Figure 2 displays SER scores and the corresponding confidence intervals for all care providers, sorted according to their number of patients. We arranged the providers in this way because we expected that practice variation may be linked to the number of patients treated by the care provider. As could be expected, the size of the confidence intervals became smaller when the number of patients per provider increased. A SER within a confidence interval below 1, indicates that there were less observed cases than expected and that the ratio between observed and expected numbers differed statistically significantly from 1, while a SER within a confidence interval above 1, indicates vice versa that there were more observed cases than expected. It turns out that the 95% confidence intervals of only two providers were above 1, while none were below 1.

To evaluate the presence of practice variation, we tested the null hypothesis that all SERs are equal to 1 using the corresponding chi-square test. This resulted in a nonrejection of the null hypothesis (χ^2^(21) = 26.4, *p*-value = 0.19), indicating that no evidence for practice variation was found.

## Discussion

In this paper, we aimed to disentangle factors associated with discontinuity of care among a large cohort of patients with schizophrenia in the Netherlands. We included 9194 patients from the records of the largest Dutch health insurance company. With this data, we were able to reconstruct the care received before the elective psychiatric care in December 2014–January 2015 and to prospectively test a series of putative predictors of the risk of discontinuity of care. Of all patients with schizophrenia who were receiving elective psychiatric care around 1-1-2015, 12.9% showed discontinuity of such care in 2016. This is less than an expected 16% based on the data from a previous cohort [21]. There are some differences between the two studies which have potential effects on discontinuity. The previous cohort over 2008–2011 was a mix of patients who did and did not receive elective care in a time with few co-payments. The present cohort contained only patients, who received elective psychiatric care, in a time when much higher co-payments were due. Elective care coincides with less discontinuity, and higher co-payments coincides with more discontinuity [21, 25]. We know of no other factors that may be responsible for this difference in the risk of discontinuity of care between the earlier and the current study.

In the current study, we found that the risk of discontinuity of care was higher in patients between the ages of 18 and 26, or who had more erratic patterns of care use before inclusion. Erratic patterns of care were defined as any or all of the following care: receiving less elective psychiatric care; receiving acute psychiatric care; receiving more quarters with elective care without antipsychotic medication; or receiving no elective treatment at all. Sex, urbanization, and alcohol or opioid dependency were not significantly related to discontinuity. Therefore, our findings show that the pattern of previous care consumption is an important prognostic factor of future care use. Although this should not be a surprise, it is important that previous care patterns are known when a patient is treated. We propose that previous care consumption can be used to design strategies to improve treatment retention and focus resources on those most at risk of dropping out.

Psychiatric care providers showed some practice variation in discontinuity of care, but after adjusting for all available prognostic, demographic, and care characteristics, solid evidence for practice variation could not be established. This was surprising, as we expected that practice variation would make a significant contribution to the retention of patients in mental healthcare. We know of no other studies conducted in the Netherlands to compare our findings with, but the findings speak to the success in organizing similar levels of quality of care for patients with severe mental illness throughout the Netherlands.

### Strengths and limitations

We consider the following aspects as strengths of our research. Healthcare insurance registry data are relevant, complete, relatively unbiased, and allow a substantial sample size [44, 45]. We were able to analyze the influence of practice variation on discontinuity of psychiatric care between providers because we had a large group of providers each having enough patients in our register. Because each provider had at least 50 patients, they had enough experience to provide appropriate continuous psychiatric care, which is often complicated for these patients. We were able to correct for patient characteristics and their psychiatric care history of the previous two years.

However, we also have to acknowledge several limitations of our study.

*First*, its naturalistic study design is open to bias due to the selection of patients or providers. We selected patients who were treated in specialized mental health care in the two months around 1-1-2015 and who had been treated before in 2013–2014. Therefore, we missed patients with schizophrenia who had been treated in 2013–2014 but were not treated in the two months around 1-1-2015, or who were diagnosed before 2013 but had not been treated for 2 years, or patients who had avoided treatment at all. Patients choosing Zilveren Kruis as their health insurer may deviate from other Dutch patients. Patients receiving specialized mental healthcare are unevenly distributed over Dutch health insurance companies [46]. Providers with less than 50 patients with schizophrenia and therefore less experience in providing psychiatric care for these patients might show different results.

*Second*, other unknown factors than patient and care characteristics may have influenced discontinuity of psychiatric care. Although the use of healthcare insurance registry data is a strength, the records provide no detailed information on the clinical aspects of patients and the care they received. It is highly likely that both clinical and environmental factors moderate the effects on continuity of care. Patients with chronic (or even treatment resistant) schizophrenia appear to have a different etiology and a combination of risk factors compared to patients with non-chronic schizophrenia; however, this study is unable to make this distinction. Research using more clinical and environmental information will be important to assess differences in the chance that subgroups of patients with schizophrenia discontinue psychiatric care, for instance, subgroups of patients with a first episode, chronic patients or treatment resistant patients. With more detailed information, for instance, from patient records, practice variation might be demonstrated, especially when these other characteristics differentiate patients into groups with high and low chances of discontinuity and when these groups were unevenly distributed over providers. Further research using detailed patient and care information is recommended.

*Third*, our follow-up period of 1 year is relatively short. In a study with a longer follow-up period, different associations may emerge between continuity of care and patients’ care characteristics or practice variation.

## Conclusions

Discontinuity of elective psychiatric care in patients with schizophrenia who were receiving elective psychiatric care at baseline occurred in 12.9% of the patients. We found evidence for an association between previous care characteristics and discontinuity of psychiatric care during the one-year follow-up period in patients with schizophrenia. This is important because previous research [21, 25] showed that discontinuity of psychiatric care is associated with more psychiatric crisis care and hospitalization. Therefore, we propose that previous care consumption can be used to stratify strategies to improve treatment retention and focus resources on those most at risk of dropping out.

The risk of discontinuity of care was higher in younger patients (age between 18 and 26), patients with a history of receiving less elective psychiatric care, more acute psychiatric care, relatively more elective care without antipsychotic medication, or no elective treatment at all.

We could not demonstrate that practice variation between providers influenced treatment discontinuity. Further research, using more detailed clinical and care characteristics in relation to discontinuity of treatment is recommended.

## Data Availability

The registry data that were used in this study are not publicly available due to Dutch privacy laws and regulations.

## Abbreviations

DBC: Diagnoses treatment system
ATC: The Anatomical Therapeutic Chemical (ATC) classification system
DSM-IV: Diagnostic and Statistical Manual of Mental Disorders-IV
AIC: The Akaike information criterion
SER: Standardized event ratio
